# Population Genetic Differences along a Latitudinal Cline between Original and Recently Colonized Habitat in a Butterfly

**DOI:** 10.1371/journal.pone.0013810

**Published:** 2010-11-03

**Authors:** Sofie Vandewoestijne, Hans Van Dyck

**Affiliations:** Biodiversity Research Centre, Earth and Life Institute, Université catholique de Louvain, Louvain-la-Neuve, Belgium; American Museum of Natural History, United States of America

## Abstract

**Background:**

Past and current range or spatial expansions have important consequences on population genetic structure. Habitat-use expansion, i.e. changing habitat associations, may also influence genetic population parameters, but has been less studied. Here we examined the genetic population structure of a Palaeartic woodland butterfly *Pararge aegeria* (Nymphalidae) which has recently colonized agricultural landscapes in NW-Europe. Butterflies from woodland and agricultural landscapes differ in several phenotypic traits (including morphology, behavior and life history). We investigated whether phenotypic divergence is accompanied by genetic divergence between populations of different landscapes along a 700 km latitudinal gradient.

**Methodology/Principal Findings:**

Populations (23) along the latitudinal gradient in both landscape types were analyzed using microsatellite and allozyme markers. A general decrease in genetic diversity with latitude was detected, likely due to post-glacial colonization effects. Contrary to expectations, agricultural landscapes were not less diverse and no significant bottlenecks were detected. Nonetheless, a genetic signature of recent colonization is reflected in the absence of clinal genetic differentiation within the agricultural landscape, significantly lower gene flow between agricultural populations (3.494) than between woodland populations (4.183), and significantly higher genetic differentiation between agricultural (0.050) than woodland (0.034) pairwise comparisons, likely due to multiple founder events. Globally, the genetic data suggest multiple long distance dispersal/colonization events and subsequent high intra- and inter-landscape gene flow in this species. Phosphoglucomutase deviated from other enzymes and microsatellite markers, and hence may be under selection along the latitudinal gradient but not between landscape types. Phenotypic divergence was greater than genetic divergence, indicating directional selection on some flight morphology traits.

**Main Conclusions/Significance:**

Clinal differentiation characterizes the population structure within the original woodland habitat. Genetic signatures of recent habitat expansion remain, notwithstanding high gene flow. After differentiation through drift was excluded, both latitude and landscape were significant factors inducing spatially variable phenotypic variation.

## Introduction

Range expansions are recurrent events that have important genetic consequences [1]. Historical range expansions (and contractions) are mainly associated with post-glacial recolonization events and are thought to be the principal factor influencing the genetic population structure in many species [2]. Currently, range expansions are increasing in frequency and rate due to climate change [3] and in several cases also to human-mediated introductions [4]. Consequences of range expansion on genetic diversity and genetic population structure are important for understanding evolutionary processes, for example to distinguish between selection and drift. Many genetic patterns previously attributed to distinct selective processes, may also result from the dynamic nature of a species range [1]. Recently colonized populations typically display lower genetic diversity and higher genetic differentiation due to repeated bottlenecks. Bottlenecks may decrease the evolutionary potential of species [5] which may then influence their capacity to adapt to heterogeneous and changing environmental conditions. However, besides range expansion, altering habitat associations or habitat expansion may also enable organisms to cope with changing environments. Climate change may partly explain altered species-habitat associations [6]. Oliver et al. [7] showed, for example, that geographic variation in habitat association with significant changes in habitat specificity at range boundaries in British butterflies. The genetic consequences of this type of expansion have only rarely been addressed.

In this study, we used microsatellite loci to study the genetic consequences of range and habitat expansion in the speckled wood butterfly, *Pararge aegeria*. The range of *P. aegeria* has shifted to the north with recent climate change [8]. Contrary to many other butterfly species, *P. aegeria* has recently increased both in distribution and abundance within the core part of its European range (including The Netherlands and Belgium)[9]. This is accompanied by an expansion in habitat-use from woodlands to more open anthropogenic landscape, like agricultural land with hedgerows [10]. Agricultural landscapes differ from woodlands both in microclimatic conditions relevant for flight [10] and the degree of fragmentation of resources, as resources are much more scattered in the agricultural landscape. Populations originating from woodland landscape differ from populations of agricultural landscape in several morphological, behavioral and life history traits [11]–[13]. Some of the differences point to genetic adaptation [14], whereas others to phenotypic plasticity [15], [16].

**Figure 1:**
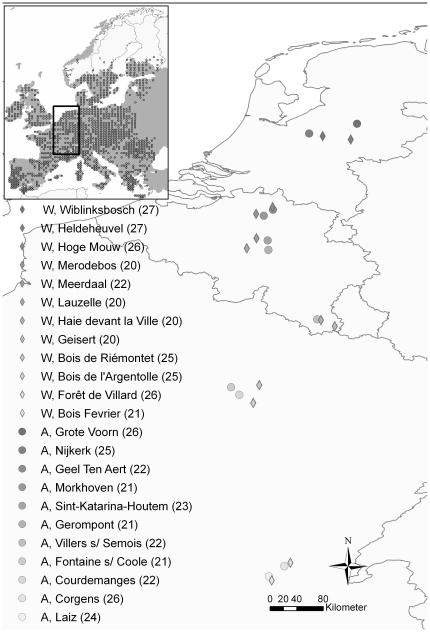
*Pararge aegeria* sampling sites. Landscape: woodland (◊, W) and agriculture (**•**, A). Sample sizes are indicated between brackets after the population name. Insert: modeled climate space (shaded area), distribution (circles) of *Pararge aegeria*, Source: reproduction with kind permission by J. Settele [71].

The overall aim of the present study is to analyze the population genetic structure in relation to expansion in habitat use. Specifically, we aimed for analyzing and comparing the genetic population structure of *P. aegeria* in permanent woodland populations and recently colonized populations in agricultural landscapes (Figure 1) to address the following questions: (*i*) how are woodland populations structured in space along the latitudinal cline, i.e. is there a genetic signature of post-glacial recolonization?; (*ii*) is effective connectivity between *P. aegeria* populations high?; (*iii*) how were the agricultural populations colonized, i.e. from one southern agricultural source population, or several independent colonization events from agricultural and/or woodland populations?; (*iv*) is there directed gene flow through matching habitat choice (woodland versus agricultural landscape) [17]? Furthermore, we test for spatially variable selection by comparing the degree of differentiation in quantitative traits with the degree of differentiation that could be generated by drift alone. Clear geographic patterns in flight-related morphological traits were observed in this species along a latitudinal cline and between landscapes [18]. Neutral genetic variation in the same individuals was used to account for phenotypic variation caused by drift. Finally, we used allozymes to test for candidate loci. As numerous studies have shown selection on allozymes in response to habitat heterogeneity in butterfly species (e.g. [19], [20]). We tested for similar patterns in *P. aegeria* relative to latitude and landscape.

## Results

### Molecular marker polymorphism

All microsatellite loci were polymorphic with a total of 195 alleles over 6 loci and a minimum of 23 alleles for *Pae3* and a maximum of 46 alleles for *Pae7*. Allozyme loci were less polymorphic, with a total of 32 alleles for 4 loci.

After controlling for the false discovery rate [21], no primer pair - population combinations were in linkage disequilibrium. *Pae3*, *Pae4* and *Pae11* showed significant Hardy-Weinberg disequilibrium due to heterozygote deficit in global tests (Table S1). All but two population – primer pairs had null allele frequencies >0.2 (population C2 – *Pae3* (0.233) and population F1 – *Pae11* (0.2192)). However, null allele frequency estimates were relatively low (0.0016–0.0843, Table S1). Below 0.20 simulation studies have shown that the bias introduced by null alleles is negligible [22]. Additionally, analyses taking null allele frequencies into account [23] gave similar results (details not shown). Consequently, we only discuss the results from the original data set.

After controlling for false discovery rate [21], neither significant linkage disequilibrium nor departures from HW were detected with the allozyme data.

Population differentiation for PGM was much higher than expected (probability of simulated values as small as or smaller than observed data, *P* >0.998), suggesting some signature of selection. No other loci showed signatures of selection as a rerun of the same analysis without PGM ensured. Therefore, the PGM locus was excluded from all subsequent analyses.

### Genetic diversity

**Figure 2:**
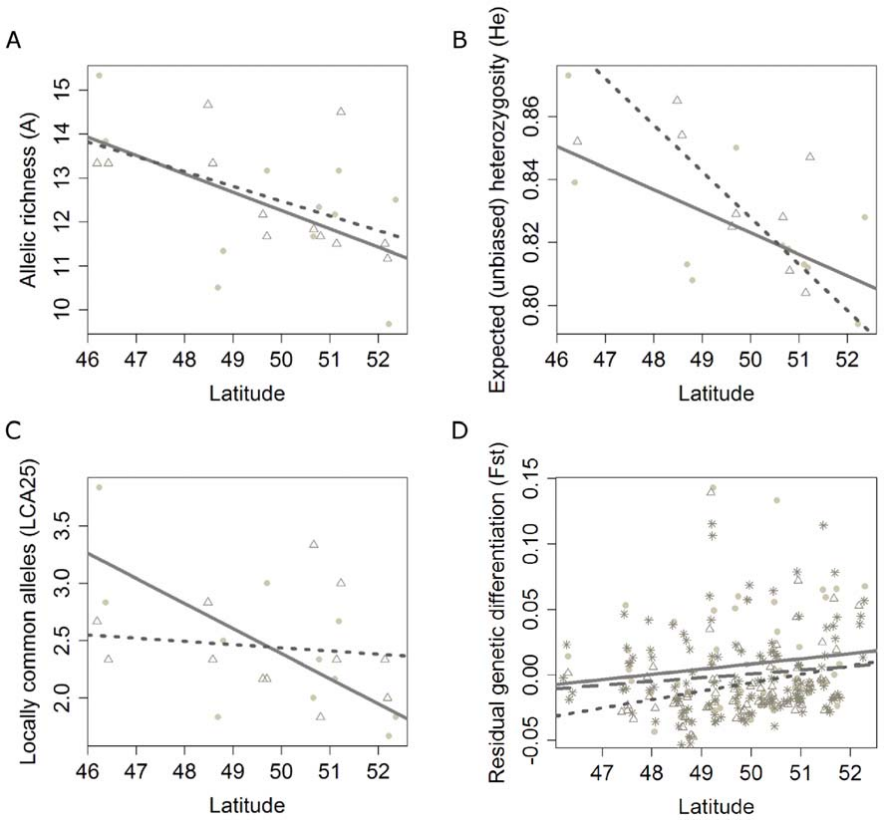
Genetic diversity and differentiation relative to latitude and landscape. (A) expected heterozygosity, (B) allelic richness, (C) locally common alleles (25%). Full line: linear regression for agricultural populations (•), discontinuous line: woodland populations (Δ). Significance levels are available in Table S3. (D) Residual population genetic differentiation. Full line: linear regression for agricultural-agricultural (•), dotted line: woodland-woodland (Δ), discontinuous line: agricultural-woodland (*) pairwise comparisons.

Genetic diversity based on microsatellites was high (Table 1, for population based estimates see Table S2). Unbiased expected heterozygosity, allele richness and LCA25 decreased with latitude, but there were no significant differences between the landscapes (Figure 2, Table S3). Slightly non-significant (*P* = 0.054) latitude x landscape interaction effects were detected, suggesting that the decrease in diversity with latitude was not completely equivalent for the two landscapes (Figure 2). Sample size had no effect on the diversity measures used. No significant bottlenecks were detected in the agricultural landscape.

**Table 1:** Genetic diversity and differentiation in *P. aegeria* (bold) and other species, based on microsatellites and allozymes.

	Microsatellite data			
	UH_e_	A	F_st_ W&C	F_st_ RH'
	mean (SD)	mean (SD)	mean (95% CI)	
***Pararge aegeria*** ** (>700 km)**				
**Agriculture**	0.825 (0.022)	12.333 (0.946)	0.013 (0.009–0.016)	0.04236
**Woodland**	0.830 (0.035)	12.556 (1.451)	0.009 (0.004–0.014)	0.01758
**Total study**	0.828 (0.029)	12.449 (1.371)	0.011 (0.008–0.014)	0.023
*Erynnis propertius* (>2000 km) [28]	0.709–0.903	3.5	0.058–0.070	
*Papilo zelicaon* (>2000 km) [28]	0.432–0.866	4.5	0.040–0.051	
*Speyeria idalia* (>2000 km) [27]	0.852–0.939	16.15–22.65	0.015–0.049	
*Polyommatus bellargus* (regional scale) [30]	0.64–0.72		0.127	
*Lycaena helle* (regional scale) [31]	0.69 (0.02)	5.35 (0.47)	0.137	
*Melitaea cinxia* (regional scale) [72]	0.42–0.89		0.060	
	Allozyme data (without PGM/*with PGM*)			
	UH_e_	A	F_st_	F_st_ RH'
	mean (SD)	mean (SD)	mean (95% CI)	
***Pararge aegeria*** ** (>700 km)**				
**Agriculture**	0.065 (0.030) /*0.096 (0.034)*	2.152 (0.565) /*2.409 (0.478)*	0.006 (−0.002–0.008) /*0.028 (0.003–0.045)*	0.005/*0.036*
**Woodland**	0.047 (0.035) /*0.086 (0.056)*	1.778 (0.643) /*2.250 (0.631)*	0.008 (0.008–0.010) /*0.036 (0.008–0.053)*	0.015/*0.028*
**Total study**	0.056 (0.034) /*0.091 (0.046)*	1.957 (0.638) /*2.326 (0.556)*	0.007 (0.001–0.008) /*0.030 (0.006–0.046)*	0.008/*0.028*
*Pararge aegeria* (< 300 km) [32]	0.05–0.12	1.2–1.9		
*Pararge aegeria* (25 km) [11]	0.30–0.40	2.5	0.018	
*Maniola jurtina* (3500 km) [73]	0.172	2.68	0.034	
*Maniola jurtina* (900 km) [73]			0.013–0.025*	
*Aglais urticae* (1000 km) [74]	0.248	2.840	0.030	
*Melanargia galathea* (1000 km) [26]	0.411	3.190	0.034	
*Melanargia galathea* (200 km) [75]			0.048	

UHe: unbiased expected heterozygosity, A: allelic richness, F_st_ W&C and F_st_ RH': genetic differentiation calculated according to [24] and [59] respectively with standard deviation (SD) or 95% confidence intervals (95% CI). For allozyme data in *P. aegeria*, results without PGM (normal case) and with PGM are given (italic case). Geographic scales are indicated between brackets after species name.

Allozyme based genetic diversity was relatively low, especially for heterozygosity estimates (Table 1). Genetic diversity also decreased with latitude for this molecular marker (Table S4). Contrary to similar levels of diversity between landscapes with microsatellite data, observed heterozygosity was significantly higher in the agricultural landscape compared to the woodland landscape for allozyme data based on three loci only (Figure S1).

### Population structure and dynamics

Population differentiation was statistically significant but very small (Table 1), with F_st_ *sensu* Weir-Cockerham [24] for all populations 0.011 (0.0075–0.0140), with slightly greater differentiation for agricultural population pairs (0.013, CI: 0.0094–0.0161) compared to woodland pairs (0.009, CI: 0.0041–0.0149). For weak differentiation, more unbiased estimates with low variance were obtained following Raufaste and Bonhomme (2000). F_st_ *sensu* Raufaste-Bonhomme (F_st_ RH') for all populations was 0.041 (CI:0.0359–0.0451), and was larger for agricultural populations (0.050, CI:0.0381–0.0615) than for woodland populations (0.034, CI: 0.0254–0.0419). While genetic differentiation was significantly higher between agricultural than woodland pairwise comparisons (F_1,117_ = 4.821, *P* = 0.030), the degree of inter-landscape differentiation was significantly different neither from agricultural - agricultural (F_1,183_ = 1.7965, *P* = 0.181) nor woodland – woodland population comparisons (F_1,194_ = 2.5136, *P* = 0.115). Population genetic differentiation increased (F_1,247_ = 6.450, *P* = 0.013) with latitude. The latitude x landscape interaction effect was not significant, i.e. population differentiation varied in a similar way along the latitudinal cline for both landscapes.

Genetic similarity decreased with increasing distance in both the agricultural and woodland landscape samples, based on Mantel tests and spatial autocorrelation analyses (Figure S3). Significant isolation by distance was observed for all population pairwise comparisons (r^2^ = 0.377, *P* = 0.003), as well as for agriculture-agriculture population pairs (r^2^ = 0.337, *P* = 0.041) and woodland-woodland population pairs (r^2^ = 0.461, *P* = 0.003). Genetic similarity fell to zero at a smaller distance interval in the agricultural landscape (61–137 km class ) than in the woodland landscape (137–195 km), indicating a larger neighborhood size for woodlands (considered to be a basic population unit, defined as a product of population density and parent–offspring dispersal distance [25]). Spatial structure was similar between landscapes at short distances, i.e. the highest autocorrelation coefficients were similar between both landscapes (r = 0.12). However, at large distances, spatial structure was much higher for woodland populations (lowest r = −0.014) than for agricultural populations (lowest r = −0.009). Significant negative correlations were detected in smaller distance classes within the agricultural (270–297 km) compared to the woodland (297–476 km) landscape.

**Figure 3:**
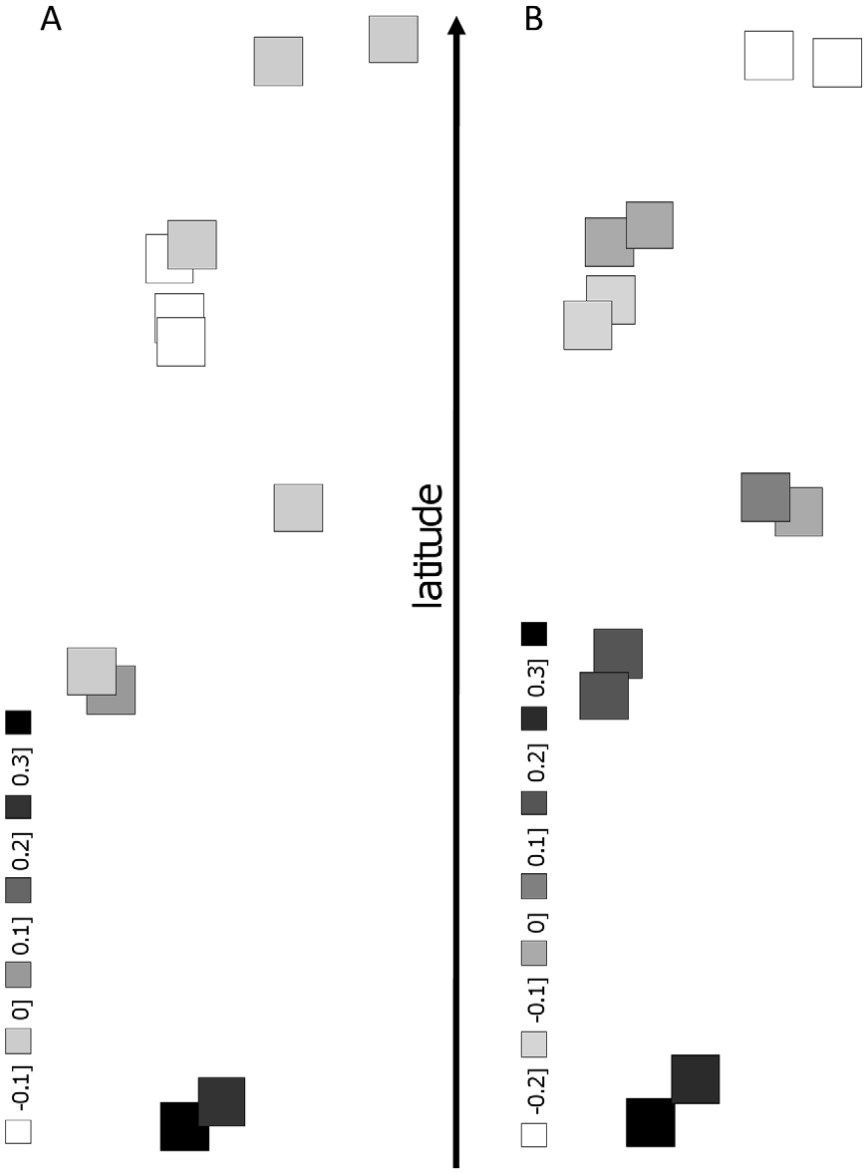
Spatial multivariate analysis. Squares represent first axis PCA scores of the A) agricultural and B) woodland populations and are placed according to their geographic coordinates. Large black squares correspond to high positive autocorrelation scores, whereas large white squares correspond to high negative scores. Gradual variation in autocorrelation scores represents clinal, isolation-by-distance genetic variation. Global scores were significant for woodland but not for agricultural populations (see text).

No clear population structure was detected by genetic clustering of populations by Bayesian inference. The highest likelihood, with or without prior geographic information, was for K = 1 in all runs. However, using spatial multivariate analyses, a significant clinal pattern was detected within the woodland landscape only (Figure 3). Only the first sPCA eigenvalue was retained, as it was strikingly large compared to all other values. The first score revealed a north-south clinal pattern associated to a strong spatial autocorrelation in woodland populations (I = 0.4470). This pattern suggests progressive genetic differentiation from one population to the other suggesting an isolation-by-distance pattern. The global structure was significant (*P* = 0.031), while the local structure was not significant in woodlands, i.e. neighbors were not genetically more different than randomly chosen pairs. Neither global nor local structures were significant in the agricultural landscape.

### Dispersal

Genetic analyses suggest lower dispersal within the woodland compared to the agricultural landscape. Using the private allele method, a significant lower number of migrants per generation Nm (*P*<0.001) was detected for agricultural populations (3.49, CI:3.300–3.688) compared to woodland populations (4.18 , CI:3.991–4.375). Low F_st_ values (see above) indicate high dispersal, with higher dispersal in the woodland compared to the agricultural landscape (higher F_st_ values for the latter). .

Maximum likelihood estimates of dispersal did not support landscape specific dispersal. Indeed, the full model, i.e. asymmetric dispersal between all populations both within landscapes and between different landscapes, had the highest likelihood (Table S5). Both the woodland source and landscape-selective dispersal models were significantly different from the full model for all latitudinal regions.

### Selection

Population differentiation in PGM decreased significantly with latitude, but no significant relation with landscape was detected (Table 2). The latitude effect remained even when variation in geographic distance was taken into account.

**Table 2:** Signatures of selection in morphology and allozymes.

	Sum. Squared		DF		F		p
**Mass (PC1)**							
latitude	0.0196	1		1.0627		0.29	
landscape	0.0882	2		2.3877		*0.093*	
latitude x landscape	0.0633	2		1.7136		0.17	
residuals	4.5617	247					
**Aspect ratio (PC4)**							
latitude	0.1674	1		7.6846		**0.002**	
landscape	0.1096	2		2.5156		*0.077*	
latitude x landscape	0.0105	2		0.2415		0.759	
residuals	5.3803	247					
**Melanisation**							
latitude	0.043	1		1.7916		0.204	
landscape	0.2088	2		4.3492		**0.007**	
latitude x landscape	0.0451	2		0.9397		0.404	
residuals	5.9295	247					
**PGM**							
latitude	0.18342	1		45.9241		**<0.0001**	
landscape	0.00297	2		0.372		0.703	
latitude x landscape	0.00155	2		0.1938		0.828	
residuals	0.98649	247					

Regression of residual variation (after considering genetic differentiation) in phenotypic population differentiation (P_st_) of size, aspect ratio and melanization to latitude, landscape and latitude x landscape. Regression of residuals of genetic differentiation F_st_ (after considering geographic distance) in PGM.

**Figure 4:**
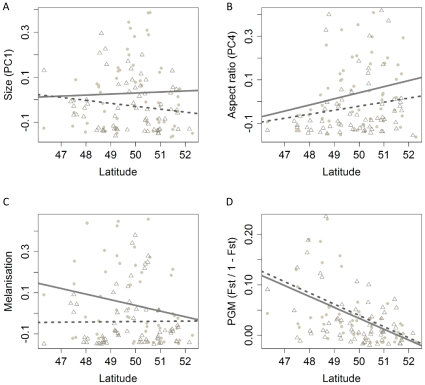
Pairwise population differentiation in relation to latitude and landscape. Residual phenotypic variation of (A) Size, (B) Aspect ratio, (C) melanization and residual genetic differentiation of (D) PGM. Full line: linear regression for agricultural-agricultural pairwise comparisons (•), dotted line: woodland-woodland pairwise comparisons (Δ). Significance levels are available in Table 2.

Residual variation of phenotypic variation (P_st_, after considering drift though variation in F_st_) in mass (PC1), relative thorax (PC4) and melanization was higher in the agricultural versus the woodland population comparisons (Table 2). Selection varied with latitude only for aspect ratio, with increased population differentiation towards the north (Figure 4).

## Discussion

High genetic diversity and weak population differentiation suggest high gene flow and/ or high population density in *P. aegeria*. A clear decrease in diversity with latitude is likely the result of post-glacial recolonization. A genetic signature of recent colonization was reflected in increased inter-population differentiation, lower gene flow and absence of clinal genetic differentiation within the agricultural landscape. Hence, our genetic data suggest multiple long distance dispersal/colonization events and subsequent high intra- and inter-landscape gene flow in this species.

### Genetic diversity and population structure of *P. aegeria* woodland populations

We observed high genetic diversity and low differentiation which is typical of high density and/or highly mobile species [26]. Microsatellite based unexpected heterozygosity and genetic differentiation were indeed similar to other butterfly species characterized either by high gene flow such as *Speyeria idalia* [27], or strong fliers and resource generalist species such as *Papilo zelicaon* [28] (Table 1). Microsatellite-based diversity was higher and genetic differentiation lower than in the case of resource specialists (Table 1), and hence species with typically lower dispersal propensity [29], such as *Polyommatus bellargus* [30] or *Lycaena helle* [31]. Allozyme-based diversity estimates and genetic differentiation were, however, much lower compared to studies at a comparable spatial scale (Table 1). This inter-marker discrepancy may be due to predominance of one allele at each locus (mean number of polymorphic loci at the 95% level, P_95%_: 0.364±0.131) for *P. aegeria* in this study. This type of variation was observed in the same species in a preliminary study by our group with low H_e_ (0.056) and predominance of one allele (P_95%_: 0.300) for 10 polymorphic loci (Vande Velde, Vandewoestijne & Van Dyck, unpublished data). In their study on *P. aegeria*, Hill *et al.* [32] were able to reveal 6 polymorphic loci. Expected heterozygosity was similar to the values in our study including PGM. Berwaerts *et al.* [11] on the other hand obtained very high expected heterozygosity estimates with only two loci for the esterase enzyme (for which we were unable to obtain clear banding patterns). This enzyme may be under selection, as PGM most likely is in our study (with an average H_e_ of 0.198 for this locus alone). The low level of allozyme polymorphism observed in *P. aegeria* can be interpreted within an evolutionary context. It is a phenotypically plastic species both for morphology and life-history traits [33]–[35], and may not have to rely on genetic polymorphisms for enzymes to survive and reproduce successfully in different environments. This hypothesis needs further investigation.

Both microsatellite and allozyme genetic diversity decreased with latitude. This is most likely due to successive founder effects and typical of post-glacial recolonization events [2]. Hill et al. [32] also observed this effect in recently expanded woodland populations of *P. aegeria*. However, increase in environmental heterogeneity with latitude should not be ruled out as Excoffier *et al.* [1] demonstrated that decrease in genetic diversity and increased genetic differentiation could also result from increased environmental heterogeneity. Increased differentiation with latitude is often detected because populations are smaller and more isolated towards the edge of their range. However, this hypothesis can be refuted here, as the most northern samples of the current study correspond to the core range of *P. aegeria tircis*.

Multivariate spatial analyses showed a clinal population genetic structure for the woodland landscape. This is likely the result of step-by-step dispersal movements. Significant isolation by distance also confirmed the genetic proximity of geographically closely located populations, and genetic distinctiveness of remote populations.

Population differentiation (F_st_) for both microsatellite (F_st_ W&C: 0.009, F_st_ RH': 0.018) and allozyme (F_st_ W&C: 0.008, F_st_ RH': 0.015) data was extremely low and highly similar between markers when the “selected” enzyme (PGM) was excluded from the analysis. Compared with other butterfly species (Table 1), differentiation is low even when considering studies at much larger scales, suggesting high effective dispersal in *P. aegeria*.

### Colonization of agricultural landscape

Neither a decrease in genetic diversity in the recently colonized landscape nor a disproportional decrease in allelic richness compared to heterozygosity was observed in *P. aegeria*. This suggests that there remains little or no genetic effects of recent colonization of the agricultural landscape, unlike other studies such as the colonization of the urban areas by the blackbird *Turdus merula* [36]. High gene flow following colonization is one likely explanation, although insufficient variation (loci) or homoplasy of microsatellites at large geographic scales [37] may also impede the detection of local population structure. However, several results suggest that the agricultural landscape was colonized, amongst others, by long distance dispersal events. Indeed, spatial multivariate analyses found no clear genetic cline (absence of global structure) in the agricultural landscape, although clinal differentiation was clearly present in the woodland landscape. Higher differentiation (F_st_) between agricultural populations also supports this hypothesis. First of all, during the colonization period, F_st_ increases because the number of demes is increasing and because the migrants founding the new demes have less and less variability, so new demes differ more from the average deme [38]. Secondly, Bialozyt *et al.* [39] found that propagation of genetic variants far away from their place of origin could result in locally reduced genetic diversity by founder effects (i.e. in this study, lower diversity towards the north), but regionally high variation (F_st_), i.e. higher differentiation towards the north as observed in this study. Subsequent gene flow will probably homogenize population structure, resulting in decreased F_st_ values, similar to those observed within the woodland landscape. The absence of significant differences in population differentiation between agricultural – woodland population pairs with both within-landscape pairs (agriculture-agriculture and woodland-woodland) suggest that colonizers originated from both agricultural and woodland populations.

Finally, weaker isolation by distance and statistically significant negative correlations at shorter distance in the agricultural landscape (spatial autocorrelation analyses) also suggest colonization through long distance dispersal in the agricultural landscape. Although isolation by distance is generally interpreted as equilibrium between drift and migration, it may also be the consequence of serial founder effects accompanying range expansion from one or several agricultural populations [40]. Increased population structure without reduced genetic diversity in recently colonized areas compared to native range was also explained by long-range dispersal of genetically distinct propagules across the introduced range in *Centaurea diffusa* [41].

However, greater extinction-recolonization dynamics within the less buffered agricultural landscape may also contribute to higher population differentiation. Nevertheless, numerous extinctions and recolonizations should also result in decreased genetic diversity within the agricultural landscape, and no significant landscape differences were detected.

### Gene flow in P. aegeria

Dispersal is a function of dispersal propensity during emigration, displacement during transfer and settlement during immigration [42]. Dispersal capacity is an individual rather than species-specific trait, i.e. high intra-specific variability in dispersal is common among European butterflies[43]. Behavioral studies on *P. aegeria* have shown landscape related variation for both dispersal propensity and immigration. In an experimental landscape with lab-reared individuals, Merckx et al. [12] showed that woodland individuals were more willing to fly and to cross open-shade boundaries than agricultural individuals, i.e. dispersal propensity is higher in woodland butterflies likely due to increased boundary permeability. Observed differences in habitat detection ability relate to the differential spatial resource grain of woodland and agricultural landscapes for *P. aegeria* [44]. Population differentiation (F_st_) and the private allele based dispersal estimates confirm more migrants between woodland compared to between agricultural populations. On the other hand, speckled woods of agricultural populations are able to target habitat from a wider distance than woodland individuals [44]. Hence, settlement success is likely to be greater due to much wider perceptual ranges. Consequently, they may be more successful at dispersing over long distances. Our results on spatial autocorrelation suggest that woodland butterflies disperse farther than agricultural butterflies, i.e. larger neighborhood size, although higher population density may give similar results. Even though the only estimate of population density indicated greater density in the agricultural landscape [13], this does not necessarily reflect higher effective population size as butterflies in the woodlands are less concentrated per unit of habitat surface than in the agricultural landscape (Vandewoestijne & Van Dyck, personal observations). Therefore, we argue that our results reflect long-term step-by-step dispersal in the woodland landscape, and recent long-distance dispersal events for the agricultural landscape. As agricultural populations are relatively recent, the process of step-by-step dispersal has not yet erased the initial effects of long distance colonization events [45].

The absence of habitat specific dispersal suggests that the more northern agricultural populations may have been established through dispersal from both woodland and other agricultural populations. The results also suggest that there is no evidence for habitat-directed dispersal in *P. aegeria*.

### Selection in relation to landscape and latitude

Phenotypic differentiation (P_st_) for forewing size, forewing aspect ratio and basal wing melanization, was significantly larger than genetic differentiation. This suggests that the degree of differentiation in quantitative traits exceeds the differentiation by genetic drift alone. Directional selection favoring different phenotypes in different populations is plausible [46]. By using phenotypic differentiation, we cannot rule out other potential causes of phenotypic variation, such as non-adaptive phenotypic plasticity or maternal effects. However, results of meta-analysis [46] suggest that information from wild phenotypes does not tend to yield higher estimates than common garden experiments. Also, the traits measured are directly related to flight performance and thermal regulation, hence their variation can be interpreted within an adaptive framework [47].

Differentiation in aspect ratio increased with latitude. Differentiation was higher amongst agricultural populations than woodland populations for mass, aspect ratio and melanization. Since variation in forewing aspect ratio is tightly related to mate-locating behavior in this butterfly, increased differentiation with latitude in this trait may suggest increased selection on mate-locating strategy. This may be indirectly related to temperature (decreased temperature with increased latitude), as habitat structure related thermal conditions influence the ratio of alternative mate-locating strategies (i.e. aggressive perching sit and wait strategy on a sunlit patch versus a searching patrolling strategy [48].

Smaller differentiation towards the north suggests a relaxation in selection on PGM with latitude. PGM is related to flight performance, and selection in relation to altitude in this enzyme has been observed in other butterfly species [49]. Interestingly, differentiation in melanization also tended to decrease with latitude, especially within the agricultural landscape. Particularly warm conditions at the southern range limit of this sub-species may exert selection on this enzyme and melanization in relation to overheating stress. This would especially be true within the agricultural landscape which already benefits from higher radiation [10]. To test this hypothesis, the study area should be expanded further to the north. We may expect increased selection at both the southern and northern limits of the species distribution, with relaxed selection at the center (i.e. in the most northern sampled populations of this study). Functional studies are also necessary to support the adaptive hypothesis.

Two complementary hypotheses support our observations of increased differentiation, and hence selection, between agricultural populations. Firstly, recent colonization by both short and long distance dispersal events will lead to increased population differentiation. Secondly, a more variable agricultural landscape from a thermal point of view [10] through less buffered microclimatic conditions and more frequent anthropogenic perturbations may result in increased differentiation. Future reciprocal transplant experiments should shed light on the different response mechanisms (phenotypic plasticity and/or adaptation) under different selection regimes relative to landscape and latitude.

## Materials and Methods

### Study species

The speckled wood (*Pararge aegeria* L.) primarily is a woodland butterfly, but it also occurs in fragmented, agricultural landscape with hedgerows in NW-Europe [12], [50]. The most northern agricultural populations were colonized only 10 to 15 years ago (J. Windig, personal observation). Results from a recent study [18] demonstrate that landscape, latitude and their interaction affected male adult flight morphology. Variation in adult size and the degree of wing melanization followed a classical geographic pattern, whereas flight-related morphological traits were opposite to those observed in other insects and under theoretical predictions on flight endurance under cooler conditions. Indeed, results from this study suggest that mate-location behavior may largely influence male flight morphology [18].

### Latitudinal gradient: sampled populations

Males were sampled during the summer of 2007 (August - September) and stored at −80°C. They represent a cohort of directly developed butterflies. At least 20 individuals were sampled in both agricultural (N = 11) and woodland (N = 12) populations along a latitudinal gradient of more than 700 km (Figure 1). Frozen thoraxes were used for allozyme analyses and legs were later used to extract DNA for microsatellite analyses. Morphological data were collected on the same individuals as described in [18].

### Genetic markers

Allozymes were studied following the methods described in [51]. Only three out of 14 enzymes tested revealed clear, interpretable and reproducible bands: phosphoglucose isomerase (PGI, E.C.5.3.1.9), phospoglucomutase (PGM, E.C.2.7.5.1) and glutamate oxaloacetate transaminase (GOT, E.C.2.6.1.1), resulting in 4 loci.

DNeasy Tissue Kits (QIAGEN) were used to extract genomic DNA from butterfly legs. The six polymorphic microsatellite loci used were: *Pae2*, *Pae3*, *Pae4*, *Pae7*, *Pae11* and *Pae16* [52]. Polymerase chain reactions were performed following the method described in [52]. For each marker, genotypes were scored automatically using GeneMapper 3.7 (Applied Biosystems) and manually verified and corrected in case of automatic scoring errors.

### Statistical analyses

Deviations from Hardy-Weinberg equilibrium and occurrence of linkage disequilibrium were tested by ARLEQUIN [53]. Significance levels were corrected for false positives (i.e. false discovery rate) following the procedure of Benjamini and Hochberg [21]. Results from the null allele [54] corrected genotype data set using FREENA [23] were compared to the original data set for basic analyses, giving similar results. Additionally, the highest null allele frequency observed (see results, Table S1) was in *Pae3* (0.0843). Simulations [22] showed that the bias induced by null alleles is negligible at frequencies below 0.2. Therefore, we did all analyses on the original data set. Per population allele frequencies for all allozyme and microsatellite loci are available in Table S6.

#### Neutrality of molecular markers

Loci that show unusually low or high levels of genetic differentiation are often assumed to be subject to natural selection. We tested for evidence of selection by comparing observed F_st_ values to neutral distribution of F_st_ as a function of expected heterozygosity, generated by a coalescent-based simulation model based on [55] in LOSITAN [56]. Each coalescent simulation was used to generate a total of 50,000 pair values, from which 0.995, 0.50 and 0.005 quantiles were computed.

#### Diversity

Observed and expected (unbiased) heterozygosity (H_e_), allelic richness (A) number of (private) alleles, and locally common alleles (i.e. alleles of a frequency of more than 5% occurring in less than 25% of populations) were calculated by GENALEX 6.2 [57]. Linear regressions were used to test the effect of latitude, landscape and the interaction effect on variation in genetic diversity with R 2.8.1 (R Development Core Team 2009). We tested for a disproportional decrease in allelic diversity compared to heterozygosity due to founder effects following colonization events with BOTTLENECK 1.2.02 [58].

#### Population structure and dynamics

Since genetic structure was very weak (F_st_<0.05) and loci were characterized by >2 alleles, F_st_ values were calculated following [59] to obtain an unbiased estimate with low variance by GENETIX 4.0.5.2. For all analyses F_st_ RH' was used. However, F_st_ *sensu* Weir and Cockerham [24] was also calculated to facilitate inter-study comparisons. Multiple regressions were used to test for the effect of latitude, landscape and the interaction effect on genetic population differentiation. To account for differences in inter-population distances, residuals of genetic variation after taking geographic inter-populations distances into account was used. Because of the non-independence between population pairs, a re-sampling procedure (*agricolae* package in R 2.8.1, R Development Core Team 2009) was used.

Mantel tests were used to assess the correlation between genetic and geographic distances with the *ecodist* package [60] in R 2.8.1 (R Development Core Team 2009). Significance levels were based on 10000 permutations. Spatial autocorrelation analyses were also carried out with the same package. To ensure statistical coherence, distance classes were selected so that they contained an equal number of population pairs. Under a model of restricted dispersal, it is predicted that genetic and geographic distance are positively autocorrelated at short distance, and negatively correlated at long distance.

Bayesian inference of the genetic structure was implemented with STRUCTURE 2.3.1. [61] and BAPS5 [62]. The admixture model was used to calculate the probability of individual assignments to population clusters (K) without prior information of the origin of individuals with STRUCTURE. Different numbers of population clusters (K = 1 to 23, three replicates per K) were tested to guide an empirical estimate of the number of identifiable populations. The likelihood was maximal at K = 1. Despite the use of prior information with the spatial model option in BAPS (with known geographical coordinates as the population units to be clustered) which has been shown to improve the statistical power to detect underlying population structure when the molecular data are sparse [63], the optimal number groups by far remained one for both the microsatellite and allozyme data.

Spatial multivariate analyses [64] were used to explore population structure without having to make assumptions about an underlying genetic model (sPCA) with the *adegenet* package [65] in R 2.8.1 (R Development Core Team 2009). To extrapolate the spatial pattern of genetic variability, spatial autocorrelation was added as a constraint to centered PCA scores in sPCA. Because inter-population connectivity revealed to be high, the inverse distance connection network was used. Global structures display positive spatial autocorrelation whereas local structures display negative spatial autocorrelation. Monte Carlo test enable the significance testing of global and local structures (10,000 permutations were implemented).

#### Dispersal

Bayesian inference was used to estimate recent migration rates with BAYESASS [66] using recommended settings. Non-migration rates of approximately 2/3 suggested that populations are not distinct and/or dispersal rates are very high, confirming results from other analyses. Consequently, estimated dispersal rates are not shown since it is very likely that they are underestimated using this method.

Within each latitudinal region, we tested for asymmetric dispersal between landscapes by likelihood ratio tests in MIGRATE 2.1.3. [67]. Three different models were compared by maximum likelihood estimates of theta and M: full model (dispersal rate was free to vary among all populations), woodland source model (dispersal from agricultural into woodland populations was estimated to be zero), landscape-selective dispersal model (dispersal was symmetric between populations within the same landscape and free to vary between different landscapes). The likelihood ratio test implemented in MIGRATE compares different models and tests whether they differ significantly from the full model. As start parameters, Brownian motion for microsatellite data was used, theta and M values were estimated from F_st_ calculations, Markov chain sampling: short chains 100, long chains 20. Using theta and M values of previous runs did not change the outcome of the tests.

Dispersal estimates (Nm) using the private allele method [68], implemented in GENEPOP 4.0 [69], is potentially less biased than the F_st_ island model method when using highly polymorphic markers such as microsatellites because of lower sensitivity to homoplasy [70].

#### Selection – morphology

The proportion of among population phenotypic variance in morphological traits (P_st_) was calculated as in [18] on the principal component axis which showed significantly greater differentiation than genetic differentiation (F_st_, Figure S2). We consider genetic differentiation to represent drift, and consequently test for selection in morphological traits by calculating the residual variation in P_st_ after taking variation in F_st_ into account. Residual variation was regressed against latitude, landscape and latitude x landscape to test for landscape and/or latitude dependent directional selection.

## Supporting Information

Table S1 Hardy-Weinberg equilibrium tests and null allele frequencies per locus per population. Bold numbers designate significant departure from Hardy-Weinberg equilibrium in first part of table, and a null allele frequency above 0.20 in second part of table.
(0.06 MB DOC)

Table S2 Microsatellite and allozyme genetic diversity. A: Agricultural landscape, W: woodland landscape; Ho : observed heterozygosity UHe : unbiased expected heterozygosity, A: allelic richness, PrivA: private alleles, LCA25: locally common alleles (frequency >5%, present in less than 25% populations).
(0.03 MB DOC)

Table S3 Variation in genetic diversity (based on microsatellites) in relation to latitude, landscape and latitude x landscape. He : unbiased expected heterozygosity, A: allelic richness, Private A: private alleles, LCA25: locally common alleles (allele frequency >5%, present in less than 25% populations). Bold values: p<0.05, italic values: p<0.10.
(0.05 MB DOC)

Table S4 Regression of allozyme diversity against latitude, landscape and latitude x landscape. (A) all enzymes and (B) all enzymes without PGM.
(0.03 MB DOC)

Table S5 Likelihood ratio test (LRT) between several maximum likelihood based dispersal models. Full model (dispersal rate were free to vary between all populations), woodland source model (dispersal from agricultural into woodland populations was estimated as zero), landscape-selective dispersal model (dispersal was symmetric between populations within the same landscape and free to vary between different landscapes). AIC values for each model are shown. Model with the lowest AIC is the most likely model. For latitudinal zone correspondence, consult code in Table S2.
(0.03 MB DOC)

Table S6 Allele frequency data for allozyme and microsatellite markers used in this study. Please refer to Figure 1 and Table S2 for population code.
(0.12 MB DOC)

Figure S1 Allozyme based observed heterozygosity in relation to latitude and landscape. Full line: linear regression for agricultural populations (•), dotted line: woodland populations (Δ). Significance levels are available in Table S4.
(0.02 MB TIF)

Figure S2 Genetic and phenotypic differentiation. Fst and Pst values for agricultural-agricultural (•) and woodland-woodland (Δ) population pairs of size and dispersal relevant morphological variation (relative thorax, aspect ratio, wing loading and melanization). All values are with 95% confidence intervals.
(0.16 MB TIF)

Figure S3 Spatial genetic autocorrelation correlogram of populations. Population pairs within A) agricultural and B) woodland landscapes. Dotted lines represent upper and lower 95% CI around the null hypothesis (no spatial structure). Filled dots represent significant r values (p<0.05), empty dots non-significant values. Error bars indicate 95% CI of r estimated by bootstrapping (n = 1000).
(0.15 MB TIF)
