# Supplementary material for: Population Genetic Differences along a Latitudinal Cline between Original and Recently Colonized Habitat in a Butterfly
Source: PLoS One. 2010 Nov 3;5(11):e13810. doi: 10.1371/journal.pone.0013810 (PMC2972211; doi:10.1371/journal.pone.0013810)
Supplement: Table S1 — Hardy-Weinberg equilibrium tests and null allele frequencies per locus per population. Bold numbers designate significant departure from Hardy-Weinberg equilibrium in first part of table, and a null allele frequency above 0.20 in second part of table. (0.06 MB DOC) [file pone.0013810.s001.doc]

Table S1: Hardy-Weinberg equilibrium tests and null allele frequencies per locus per population. Bold Figureures designate significant departure from Hardy-Weinberg equilibrium in first part of table, and a null allele frequency above 0.20 in second part of table.

|  |  | *Pae2* | *Pae3* | *Pae4* | *Pae7* | *Pae11* | *Pae16* |
| --- | --- | --- | --- | --- | --- | --- | --- |
| H-W test | H_o_ | 0.80524 | 0.68998 | 0.79087 | 0.77111 | 0.78571 | 0.6236 |
|  | H_e_ | 0.85265 | 0.8484 | 0.9314 | 0.77842 | 0.9335 | 0.68585 |
|  | p | 0.03994 | **p < 0.0001** | **p < 0.0001** | 0.28929 | **p < 0.0001** | 0.53124 |
|  | Population | *Pae2* | *Pae3* | *Pae4* | *Pae7* | *Pae11* | *Pae16* |
| Null allele frequencies | A1 | 0 | 0 | 0.0347 | 0 | 0.0196 | 0.0677 |
|  | A2 | 0.0441 | 0.0232 | 0.0721 | 0 | 0.0739 | 0 |
|  | A3 | 0 | 0 | 0.0458 | 0 | 0 | 0.0409 |
|  | A4 | 0.0683 | 0 | 0.0406 | 0.0016 | 0.0475 | 0.0734 |
|  | B1 | 0 | 0.0508 | 0.059 | 0 | 0.0528 | 0.0081 |
|  | B2 | 0.0781 | 0.0343 | 0.0115 | 0 | 0.0158 | 0.009 |
|  | B3 | 0 | 0 | 0 | 0.0351 | 0 | 0.0227 |
|  | B4 | 0 | 0.0654 | 0.0328 | 0.0061 | 0.1094 | 0.0945 |
|  | C1 | 0 | 0.1632 | 0.0213 | 0.0229 | 0.0272 | 0.064 |
|  | C2 | 0.0246 | **0.233** | 0.0722 | 0.0947 | 0.0717 | 0 |
|  | C3 | 0.0833 | 0.02 | 0.0497 | 0 | 0.0947 | 0 |
|  | C4 | 0 | 0 | 0 | 0 | 0.0091 | 0.0623 |
|  | D1 | 0 | 0.0255 | 0 | 0.0103 | 0.0749 | 0.1251 |
|  | D2 | 0 | 0.1601 | 0.0789 | 0 | 0.0714 | 0 |
|  | D3 | 0.0099 | 0.0449 | 0.0765 | 0.0652 | 0.0607 | 0 |
|  | E1 | 0 | 0.1343 | 0.0762 | 0.0737 | 0.0039 | 0 |
|  | E2 | 0.0864 | 0.0449 | 0.0556 | 0 | 0.0342 | 0 |
|  | E3 | 0 | 0.1417 | 0.0444 | 0 | 0.1814 | 0.0336 |
|  | E4 | 0 | 0.1024 | 0.0625 | 0 | 0.0226 | 0.0027 |
|  | F1 | 0 | 0.1626 | 0.1144 | 0.0167 | **0.2192** | 0 |
|  | F2 | 0 | 0.0847 | 0.1444 | 0 | 0 | 0.0596 |
|  | F3 | 0.0339 | 0.1574 | 0.0744 | 0.0238 | 0.154 | 0 |
|  | F4 | 0.0368 | 0.1372 | 0.1307 | 0.0058 | 0.101 | 0.0526 |
|  | All populations | 0.0231 | 0.0843 | 0.0705 | 0.0016 | 0.0751 | 0.0349 |
