# Supplementary material for: Population Genetic Differences along a Latitudinal Cline between Original and Recently Colonized Habitat in a Butterfly
Source: PLoS One. 2010 Nov 3;5(11):e13810. doi: 10.1371/journal.pone.0013810 (PMC2972211; doi:10.1371/journal.pone.0013810)
Supplement: Table S2 — Microsatellite and allozyme genetic diversity. A: Agricultural landscape, W: woodland landscape; Ho : observed heterozygosity UHe : unbiased expected heterozygosity, A: allelic richness, PrivA: private alleles, LCA25: locally common alleles (frequency >5%, present in less than 25% populations). (0.03 MB DOC) [file pone.0013810.s002.doc]

Table S2: Microsatellite and allozyme genetic diversity. A: Agricultural landscape, W: woodland landscape; H_o_ : observed heterozygosity UH_e_ : unbiased expected heterozygosity, A: allelic richness, PrivA: private alleles, LCA25: locally common alleles (frequency > 5%, present in less than 25% populations).
