# Supplementary material for: Population Genetic Differences along a Latitudinal Cline between Original and Recently Colonized Habitat in a Butterfly
Source: PLoS One. 2010 Nov 3;5(11):e13810. doi: 10.1371/journal.pone.0013810 (PMC2972211; doi:10.1371/journal.pone.0013810)
Supplement: Table S3 — Variation in genetic diversity (based on microsatellites) in relation to latitude, landscape and latitude x landscape. He : unbiased expected heterozygosity, A: allelic richness, Private A: private alleles, LCA25: locally common alleles (allele frequency >5%, present in less than 25% populations). Bold values: p<0.05, italic values: p<0.10. (0.05 MB DOC) [file pone.0013810.s003.doc]

Table S3: Variation in genetic diversity (based on microsatellites) in relation to latitude, landscape and latitude x landscape. H_e_ : unbiased expected heterozygosity, A: allelic richness, Private A: private alleles, LCA25: locally common alleles (allele frequency > 5%, present in less than 25% populations). Bold values: p < 0.05, italic values: p < 0.10.

|  | Estimate | Std. Error | DF | F | p |
| --- | --- | --- | --- | --- | --- |
| **H_e_** |  |  |  |  |  |
| intercept | 1.1650 | 0.1354 |  |  |  |
| latitude | -0.0068 | 0.0027 | 1,19 | 31.5660 | **0.0000** |
| landscape | 0.3984 | 0.1912 | 1,19 | 0.7071 | 0.4109 |
| latitude x landscape | -0.0079 | 0.0038 | 1,19 | 4.2114 | *0.0542* |
| **A** |  |  |  |  |  |
| intercept | 33.1108 | 9.1347 |  |  |  |
| latitude | -0.4170 | 0.1832 | 1,19 | 8.4581 | **0.0090** |
| landscape | -3.8301 | 12.9028 | 1,19 | 0.1500 | 0.7028 |
| latitude x landscape | 0.0809 | 0.2589 | 1,19 | 0.0976 | 0.7582 |
| **Private A** |  |  |  |  |  |
| intercept | 2.0368 | 1.7152 |  |  |  |
| latitude | -0.0357 | 0.0344 | 1,19 | 1.9788 | 0.1757 |
| landscape | -0.1604 | 2.4228 | 1,19 | 0.0105 | 0.9195 |
| latitude x landscape | 0.0030 | 0.0486 | 1,19 | 0.0039 | 0.9510 |
| **LCA25** |  |  |  |  |  |
| intercept | 13.2909 | 3.4333 |  |  |  |
| latitude | -0.2181 | 0.0688 | 1,19 | 6.3866 | **0.0205** |
| landscape | -9.4510 | 4.8496 | 1,19 | 0.0040 | 0.9505 |
| latitude x landscape | 0.1900 | 0.0973 | 1,19 | 3.8136 | *0.0657* |
