# Supplementary material for: Population Genetic Differences along a Latitudinal Cline between Original and Recently Colonized Habitat in a Butterfly
Source: PLoS One. 2010 Nov 3;5(11):e13810. doi: 10.1371/journal.pone.0013810 (PMC2972211; doi:10.1371/journal.pone.0013810)
Supplement: Table S4 — Regression of allozyme diversity against latitude, landscape and latitude x landscape. (A) all enzymes and (B) all enzymes without PGM. (0.03 MB DOC) [file pone.0013810.s004.doc]

Table S4: Regression of allozyme diversity against latitude, landscape and latitude x landscape interaction for (A) all enzymes and (B) all enzymes without PGM.
