# Supplementary material for: Population Genetic Differences along a Latitudinal Cline between Original and Recently Colonized Habitat in a Butterfly
Source: PLoS One. 2010 Nov 3;5(11):e13810. doi: 10.1371/journal.pone.0013810 (PMC2972211; doi:10.1371/journal.pone.0013810)
Supplement: Table S5 — Likelihood ratio test (LRT) between several maximum likelihood based dispersal models. Full model (dispersal rate were free to vary between all populations), woodland source model (dispersal from agricultural into woodland populations was estimated as zero), landscape-selective dispersal model (dispersal was symmetric between populations within the same landscape and free to vary between different landscapes). AIC values for each model are shown. Model with the lowest AIC is the most likely model. For latitudinal zone correspondence, consult code in Table S2. (0.03 MB DOC) [file pone.0013810.s005.doc]

Table S5: Likelihood ratio test (LRT) between several maximum likelihood based dispersal models: full model (dispersal rate were free to vary between all populations), woodland source model (dispersal from agricultural into woodland populations was estimated as zero), landscape-selective dispersal model (dispersal was symmetric between populations within the same landscape and free to vary between different landscapes). AIC values for each model are shown. Model with the lowest AIC is the most likely model. For latitudinal zone correspondence, consult code in Table S2.

|  | Latitudinal zone | |  |  |  |
| --- | --- | --- | --- | --- | --- |
|  | A | B | C | E | F |
| Full model | 9545 | 5338 | 4595 | 8573 | 6326 |
| Woodland source model | 30776 | 32334 | 36819 | 44743 | 43476 |
| Lansdcape selective dispersal model | 9956 | 5559 | 4634 | 20096 | 19590 |
| LRT: full vs. woodland source | < 0.0001 | < 0.0001 | < 0.0001 | < 0.0001 | < 0.0001 |
| LRT: full vs. landscape selective | < 0.0001 | < 0.0001 | < 0.0001 | < 0.0001 | < 0.0001 |
