# Supplementary material for: Population Genetic Differences along a Latitudinal Cline between Original and Recently Colonized Habitat in a Butterfly
Source: PLoS One. 2010 Nov 3;5(11):e13810. doi: 10.1371/journal.pone.0013810 (PMC2972211; doi:10.1371/journal.pone.0013810)
Supplement: Table S6 — Allele frequency data for allozyme and microsatellite markers used in this study. Please refer to Figure 1 and Table S2 for population code. (0.12 MB DOC) [file pone.0013810.s006.doc]

Table S6: Allele frequency data for allozyme and microsatellite markers used in this study. Please refer to Figure 1 and Table S2 for population code.
