# Supplementary figures and images for: Population Genetic Differences along a Latitudinal Cline between Original and Recently Colonized Habitat in a Butterfly
Source: PLoS One. 2010 Nov 3;5(11):e13810. doi: 10.1371/journal.pone.0013810 (PMC2972211; doi:10.1371/journal.pone.0013810)

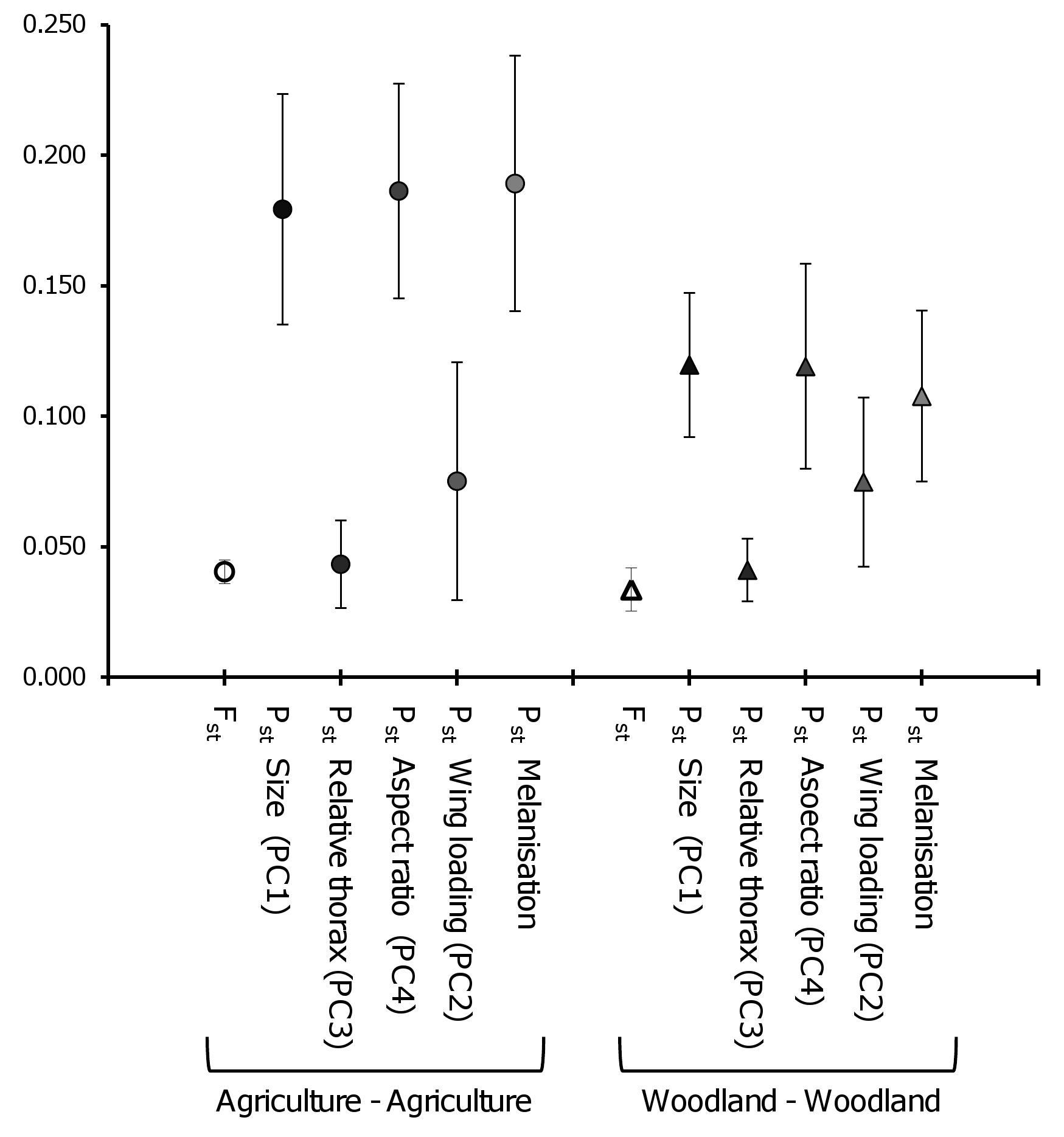

Supplement: Figure S2 — Genetic and phenotypic differentiation. Fst and Pst values for agricultural-agricultural (•) and woodland-woodland (Δ) population pairs of size and dispersal relevant morphological variation (relative thorax, aspect ratio, wing loading and melanization). All values are with 95% confidence intervals. (0.16 MB TIF) [file pone.0013810.s008.tif]

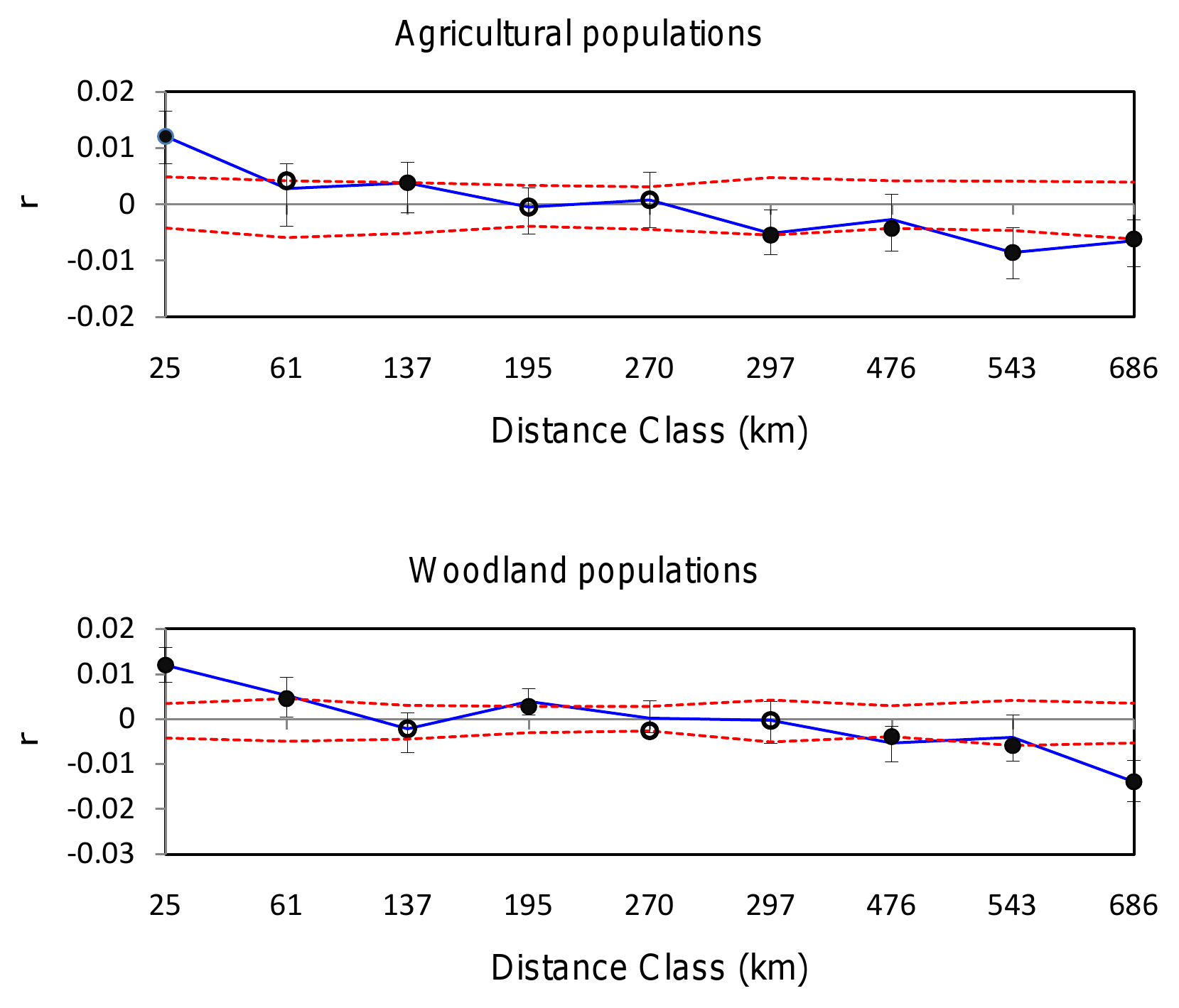

Supplement: Figure S3 — Spatial genetic autocorrelation correlogram of populations. Population pairs within A) agricultural and B) woodland landscapes. Dotted lines represent upper and lower 95% CI around the null hypothesis (no spatial structure). Filled dots represent significant r values (p<0.05), empty dots non-significant values. Error bars indicate 95% CI of r estimated by bootstrapping (n = 1000). (0.15 MB TIF) [file pone.0013810.s009.tif]
